# Marine Polysaccharide-Collagen Coatings on Ti6Al4V Alloy Formed by Self-Assembly

**DOI:** 10.3390/mi10010068

**Published:** 2019-01-19

**Authors:** Karl Norris, Oksana I. Mishukova, Agata Zykwinska, Sylvia Colliec-Jouault, Corinne Sinquin, Andrei Koptioug, Stéphane Cuenot, Jemma G. Kerns, Maria A. Surmeneva, Roman A. Surmenev, Timothy E.L. Douglas

**Affiliations:** 1 Engineering Department, Lancaster University, Lancaster LA1 4YW, UK; hwbkn3@gmail.com; 2 Physical Materials Science and Composite Materials Centre, National research Tomsk Polytechnic University, Tomsk 634050, Russia; Oksana_mishukova@mail.ru (O.I.M.); surmenevamaria@mail.ru (M.A.S.); rsurmenev@mail.ru (R.A.S.); 3 IFREMER, Laboratoire Ecosystèmes Microbiens et Molécules Marines pour les Biotechnologies, F-44311 Nantes, France; agata.zykwinska@ifremer.fr (A.Z.); sylvia.colliec.jouault@ifremer.fr (S.C.-J.); corinne.sinquin@ifremer.fr (C.S.); 4 Sports Tech Research Centre, Mid-Sweden University, Akademigatan 1, 83125 Östersund, Sweden; andrey.koptyug@miun.se; 5 Institut des Matériaux Jean Rouxel (IMN), Université de Nantes-CNRS, 44322 Nantes, France; Stephane.Cuenot@cnrs-imn.fr; 6 Lancaster Medical School, Faculty of Health and Medicine, Lancaster University, Lancaster LA1 4YW, UK; j.kerns@lancaster.ac.uk; 7 Materials Science Institute (MSI), Lancaster University, Lancaster LA1 4YW, UK

**Keywords:** marine exopolysaccharide, collagen, surface modification, Ti6Al4V

## Abstract

Polysaccharides of marine origin are gaining interest as biomaterial components. Bacteria derived from deep-sea hydrothermal vents can produce sulfated exopolysaccharides (EPS), which can influence cell behavior. The use of such polysaccharides as components of organic, collagen fibril-based coatings on biomaterial surfaces remains unexplored. In this study, collagen fibril coatings enriched with HE800 and GY785 EPS derivatives were deposited on titanium alloy (Ti6Al4V) scaffolds produced by rapid prototyping and subjected to physicochemical and cell biological characterization. Coatings were formed by a self-assembly process whereby polysaccharides were added to acidic collagen molecule solution, followed by neutralization to induced self-assembly of collagen fibrils. Fibril formation resulted in collagen hydrogel formation. Hydrogels formed directly on Ti6Al4V surfaces, and fibrils adsorbed onto the surface. Scanning electron microscopy (SEM) analysis of collagen fibril coatings revealed association of polysaccharides with fibrils. Cell biological characterization revealed good cell adhesion and growth on bare Ti6Al4V surfaces, as well as coatings of collagen fibrils only and collagen fibrils enhanced with HE800 and GY785 EPS derivatives. Hence, the use of both EPS derivatives as coating components is feasible. Further work should focus on cell differentiation.

## 1. Introduction

Metallic load-bearing implants for bone contact rely on the formation of new bone tissue on the implant surface. Modifications of the surface which promote attachment, proliferation, and osteogenic differentiation of bone-forming cells are desirable. One strategy is the coating of the surfaces with fibrils of collagen, the main structural protein of mammalian tissue. Titanium and its alloys, including Ti6Al4V, are commonly used as implant materials for load-bearing applications. Coatings of collagen types I, II, and III have been applied to improve adhesion and proliferation of bone-forming cells [1,2,3].

The collagen molecule is a long rigid complex structure consisting of three polypeptide chains which are connected to each other in the form of a triple helix configuration. Individual collagen units associate to fibrils under physiological conditions. This self-assembly of fibrils, also known as fibrillogenesis, can be induced under laboratory conditions by neutralizing an acidic solution of collagen molecules. This reduces electrostatic repulsions between the collagen molecules and enables fibrillogenesis to occur [4]. 

Collagen fibril coatings can be employed as artificial extracellular matrices into which other biologically active molecules can be incorporated. Various anionic polysaccharides, including glycosaminoglycans (GAG), are known to stimulate the attachment and proliferation of cells. Collagen fibril coatings containing GAG have been employed to improve cell adhesion and proliferation [5,6].

Recently, there has been growing interest in the use of marine polysaccharides. HE800 exopolysaccharides (EPS) is an unusual polysaccharide produced by the deep-sea hydrothermal bacterium *Vibrio diabolicus* [7]. This linear non-sulfated acidic polysaccharide is composed of a tetrasaccharide repeating unit containing N-acetyl-glucosamine (GlcNAc), two glucuronic acid (GlcA), and N-acetyl-galactosamine (GalNAc) residues [8]. HE800 EPS structure, which presents structural similarities to the GAG hyaluronic acid, confers to the EPS GAG-like properties. Native EPS of high-molecular weight (HMW) was shown to enhance in vivo bone regeneration [9] and stimulate collagen structuring by fibroblasts in reconstructed dermis [10]. GY785 EPS is a highly branched acidic heteropolysaccharide excreted by the deep-sea hydrothermal bacterium *Alteromonas infernus* [7]. This naturally slightly sulfated polysaccharide is composed of a nonasaccharide repeating unit with the main chain containing glucose (Glc), galacturonic acid (GalA), and galactose (Gal) residues. A short side chain constituted of two GlcA, Gal, and Glc is attached to a GalA residue of the main chain, bearing also a sulfate group [11]. Native HMW GY785 EPS and its low-molecular weight (LMW) chemically sulfated derivatives possess anti-coagulant [12] and anti-metastatic [13] properties, and favor chondrogenic differentiation of mesenchymal stem cells [14,15]. In summary, these EPS derivatives can inhibit some processes involved in tissue breakdown and inflammation, such as induction of matrix metalloproteases (MMP) by inflammatory cytokines (Interleukin-1β (IL-1β) and Tumor Necrosis Factor-alpha (TNF-α)) and complement cascade [10,12,13,14,15]. They can also promote in vitro cell proliferation and differentiation via major growth factors (Fibroblast Growth Factor (FGF)-2, Vascular Endothelial Growth Factor (VEGF), and Transforming Growth Factor (TGF)-β1) [11,13,14]. In similar way to heparin, EPS derivatives could also potentiate the osteogenic activities of Bone Morphogenetic Protein-2 (BMP-2) by regulating the binding to its receptors [16] or by exerting synergistic effects on osteoblasts combined with Wnt3 signaling protein involved in several development processes [17]. In contrast, they inhibit osteoclastogenesis and bone resorption. These derivatives play an important role in bone remodeling [18]. GAG-like properties of both EPS could therefore be exploited in elaboration of coatings enhancing the formation of new bone tissue on the implant surface.

In this study, Ti6Al4V samples were manufactured using an additive manufacturing method. Additive manufacturing allows the production of 3D structures with precise external dimensions and internal infrastructure, and can be used to fabricate a load-bearing implant with dimensions and architecture specifically tailored to the needs of an individual patient. The samples were subsequently coated with fibrils of collagen type I, both with and without derivatives of HE800 and GY785. The effect of the EPS derivatives on collagen fibril coating morphology and the attachment, morphology, and vitality of osteoblast-like MG63 cells was investigated. 

## 2. Materials and Methods 

### 2.1. HE800 and GY785 Exopolysaccharides (EPS) Production

Production and isolation of both EPS were previously described [7,19]. For HE800 and GY785 EPS production, respectively, *Vibrio diabolicus* and *Alteromonas infernus* were cultured in Zobell medium composed of 4 g/L of peptone, 1 g/L of yeast extract, and 33.3 g/L of aquarium salts at 25 °C and pH 7.4 in a fermenter containing 30 g/L of glucose, as a carbohydrate source. After 48 h of fermentation, the culture media were centrifuged (9000 g, 45 min), and the supernatants containing soluble EPS were ultrafiltrated on a 100 kDa cut-off membrane and freeze-dried.

### 2.2. Preparation of HE800 and GY785 EPS Derivatives

HE800 and GY785 derivatives were obtained by a free-radical depolymerization process using hydrogen peroxide, as previously described [20,21]. 

### 2.3. Characterization of EPS Derivatives

The properties of the EPS derivatives are shown in Table 1.

#### 2.3.1. Sugar Composition

Monosaccharide composition was determined according to the Kamerling et al. method [22], modified by Montreuil et al. [23]. Samples were hydrolyzed with 3 M MeOH/HCl for 4 h at 100 °C. Myo-inositol was used as an internal standard. The methyl glycosides obtained were then converted to trimethylsilyl derivatives with N,O-bis(trimethylsilyl)trifluoroacetamide and trimethylchlorosilane (BSTFA:TMCS) 99:1 (Merck). Gas Chromatography-Flame Ionisation Detector (GC-FID, Agilent Technologies 6890N, Santa Clara, CA, USA) was used to separate and quantify the per-*O*-trimethylsilyl methyl glycosides formed. 

#### 2.3.2. Molecular Weight

High-performance size-exclusion chromatography (HPSEC, Prominence, Shimadzu Co, Kyoto, Japan) coupled with multiangle light scattering (MALS, Dawn Heleos-II, Wyatt Technology, Santa Barbara, CA, USA) and differential refractive index (RI, Optilab, Wyatt technology) detectors was used to determine the weight-average molecular weight of the EPS derivatives. A refractive index increment *dn*/*dc* of 0.145 mL/g was applied to calculate the molecular weight.

#### 2.3.3. Sulfate Content

Sulfate content in the samples was quantified by high-performance anion-exchange chromatography (HPAEC) using a Dionex DX-500 (Dionex, Sunnyvale, CA, USA), as previously described by Chopin et al. [20]. 

### 2.4. Atomic Force Microscopy (AFM): Sample Preparation and Imaging

HE800 and GY785 derivatives were firstly solubilized overnight at 1 mg/mL in water and then diluted at 5 μg/mL in water. Two microliters of each diluted solution were deposited onto a freshly cleaved mica surface. Samples were then immediately dried under ambient conditions before being imaged using a NanoWizard^®^ atomic force microscope (AFM, JPK, Berlin, Germany) in intermittent contact mode at room temperature. In this imaging mode, rectangular cantilevers (Nanosensors NCL-W) with a spring constant of 40 N/m and a free resonance frequency of 165 kHz were used. The AFM tips, with a radius curvature of ~10 nm, were cleaned by UV-ozone treatment prior to AFM observation. JPK Data Processing software (JPK) was used for image processing and length measurements.

### 2.5. Production of Ti6Al4V Discs and Coating with Collagen Fibrils

The research used Ti6Al4V disks of 2 cm diameter and 2 mm thick produced using an additive manufacturing method (Electron Beam Melting, EBM) on an ARCAM EBM A2 (Arcam AB, Mölndal, Sweden) machine using the set of process parameters provided by the machine manufacturer, as described earlier [24].

Collagen fibril layers were formed by forming collagen hydrogels from acidic collagen solution, using the method of Karamachos et al. [25], on the surface of Ti6Al4V discs, as described in previous work [26]. The compositions of the hydrogels are shown in Table 2.

In brief, hydrogels were produced by mixing sterile solutions of collagen type I (BD Biosciences 354231, 4 mg/mL, San Jose, CA, USA), 10× Eagle’s Minimum Essential Medium (MEM) (M0275, Sigma–Aldrich, Saint Louis, MO, USA), and EPS derivative solution (5 mg/mL) (or ddH_2_O for control samples). Neutralization was performed by adding 2 µL increments of sterile-filtered 1 M sodium hydroxide solution until the color of the solution changed to purple (Figure S1). The purple solution was spread evenly on pre-autoclaved Ti6Al4V discs, and hydrogel formation took place at room temperature under sterile conditions for 2.5 h. Hydrogels were then removed from the surfaces of discs. Discs were rinsed three times in sterile double-deionized water (ddH_2_O) and then dried under sterile conditions in a laminar flow hood, as described previously [27].

### 2.6. Scanning Electron Microscopy (SEM) of Ti6Al4V Discs Coated with Collagen Fibrils and EPS Derivatives

Scanning electron microscopy (SEM) was performed with a JEOL (JEOL Ltd., Tokyo, Japan) in secondary electron mode at an acceleration voltage of 5 keV. Prior to SEM analysis, Ti6Al4V discs were coated with a thin layer of gold. Samples were dried prior to gold coating under sterile conditions in a laminar flow hood (see Section 2.5).

### 2.7. Cell Biological Characterization of Ti6Al4V Discs Coated with Collagen Fibrils and EPS Derivatives

#### 2.7.1. Cell Culture and Cell Seeding

The human osteosarcoma cell line MG-63 American type culture collection (ATCC) was routinely cultured in Dulbecco’s Modified Eagle Medium (DMEM) supplemented with 10% foetal bovine serum (FBS) and 1% penicillin/streptomycin. Cells were incubated at 37 °C in a humidified 5% CO_2_ environment until cultures reached 70–80% confluence. Following trypsinization, the trypan-blue exclusion assay was used to determine % viability prior to cell seeding. For all cell viability experiments, cells were resuspended in phenol-free media before a total of 3 × 10^4^ cells were seeded on to Ti6Al4V alloy discs with or without collagen or collagen-EPS derivative coatings.

#### 2.7.2. Cell Viability 

To assess whether cells were able to attach and proliferate on Ti6Al4V alloy discs with and without collagen or collagen-EPS derivative coatings, the Presto-Blue cell viability assay was performed after cells were incubated on Ti6Al4V samples for 1, 4, and 7 days. Live/Dead imaging was performed after 7 days. Prior to both procedures, cell seeded Ti6Al4V alloy discs were washed with Dulbecco’s phosphate buffered saline (DPBS) twice. The Presto-Blue cell viability reagent (ThermoFisher Scientific, Waltham, MA, USA) was diluted 1 in 10 in phenol-free DMEM before 1.5 ml was incubated with each sample for 4 h. The fluorescent signal of a 200 μL aliquot was read using an excitation of 560 nm and an emission of 590 nm. Diluted PrestoBlue reagent in the absence of cells or Ti6Al4V samples was used to measure background fluorescence which was subtracted from samples containing cells. In addition, PrestoBlue reagent was also incubated with Ti6Al4V alloy discs lacking cells to determine whether the samples interfered with the assay.

For Live/Dead imaging, cell-seeded Ti6Al4V alloy discs were incubated in DPBS containing 1 μg/mL Hoechst, 2 μM calcein-AM, and 4 μM ethidium homodimer-I for 30 min at room temperature. Individual fluorescent images were taken on an Axio Scope A1 LED microscope (Zeiss, Jena, Germany) and enhanced using ImageJ software.

## 3. Results

**Figure 1:**
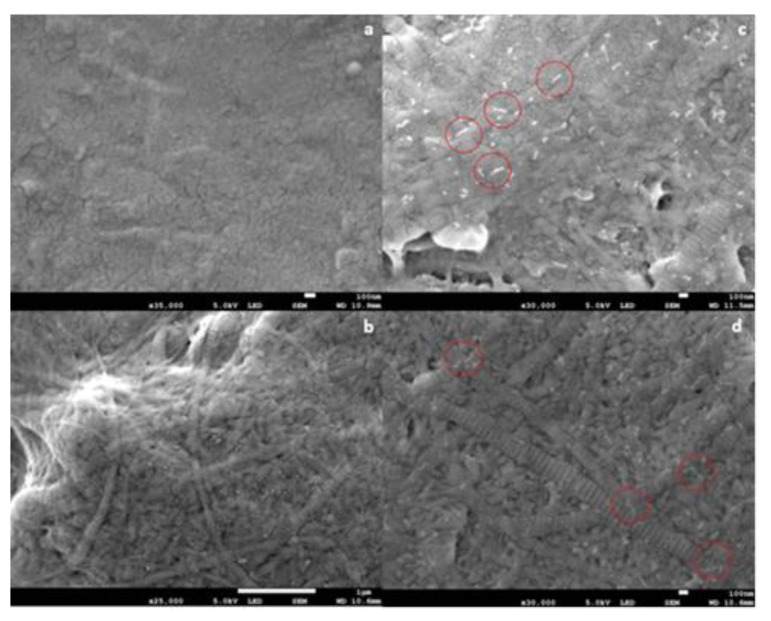
Scanning electron microscope (SEM) images of collagen fibril coatings on Ti6Al4V. (**a**) Bare Ti6Al4V; (**b**) collagen coating; (**c**) collagen + GY875 derivative coating; (**d**) collagen + HE800 derivative coating. Scale bars: (**a**) 100 nm; (**b**) 100 nm; (**c**) 1 μm; (**d**) 100 nm. Representative white “threads” on (**c**,**d**) have been indicated by the red circles.

The results of SEM analysis are shown in Figure 1. The results demonstrated the formation of collagen fibril coatings on Ti6Al4V surfaces. Fibrils exhibited a banding morphology typical for collagen. It appeared that the presence of anionic EPS derivatives increased the diameter of collagen fibrils, but this cannot be concluded conclusively from the SEM data. On surfaces coated with fibrils formed in the presence of both EPS derivatives, short “threads” were observed associated with fibrils. Such threads were absent in samples coated with pure collagen fibrils.

**Figure 2:**
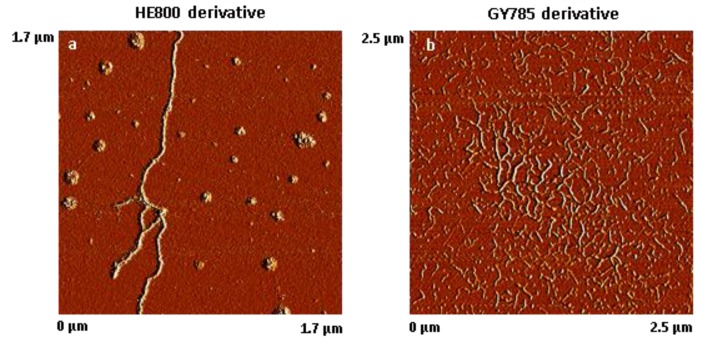
Atomic force microscope (AFM) images of dried, highly-diluted exopolysaccharides (EPS) solutions. (**a**) HE800 derivative (1.7 µm × 1.7 µm) and (**b**) GY785 derivative (2.5 µm × 2.5 µm).

The results of AFM analysis of dried, highly-diluted EPS derivative solutions are shown in Figure 2. The AFM images revealed that HE800 derivative formed threads of 702 ± 164 nm (*N* = 50) in length, which remained inter-connected due most likely to non-covalent interactions, such as water-mediated hydrogen bonds, van der Waals forces, and/or electrostatic interactions between anionic polysaccharide chains and residual ions remaining in the sample (Figure 2a). In contrast to HE800 derivative, GY785 derivative was present as short individual threads of 119 ± 31 nm in length (*N* = 80) (Figure 2b).

**Figure 3:**
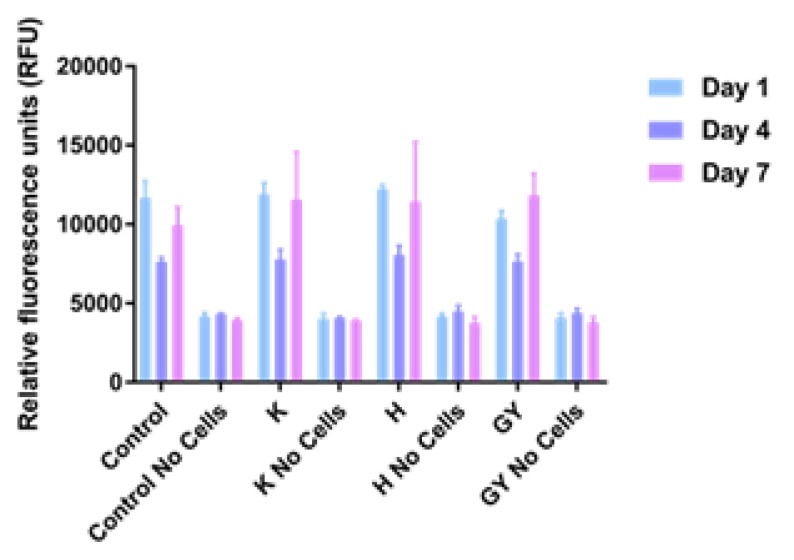
Proliferation assay of MG63 cells on Ti6Al4V samples after 1, 4, and 7 days. Error bars indicate standard deviation. Control: tissue culture polystyrene; H: collagen + HE800 derivative; K: bare Ti6Al4V; EY: collagen + GY875 derivative.

The results of cell viability studies are shown in Figure 3. Values appeared to be similar after 1, 4, and 7 days of culture on all sample types, regardless of the surface on which they were cultured. 

**Figure 4:**
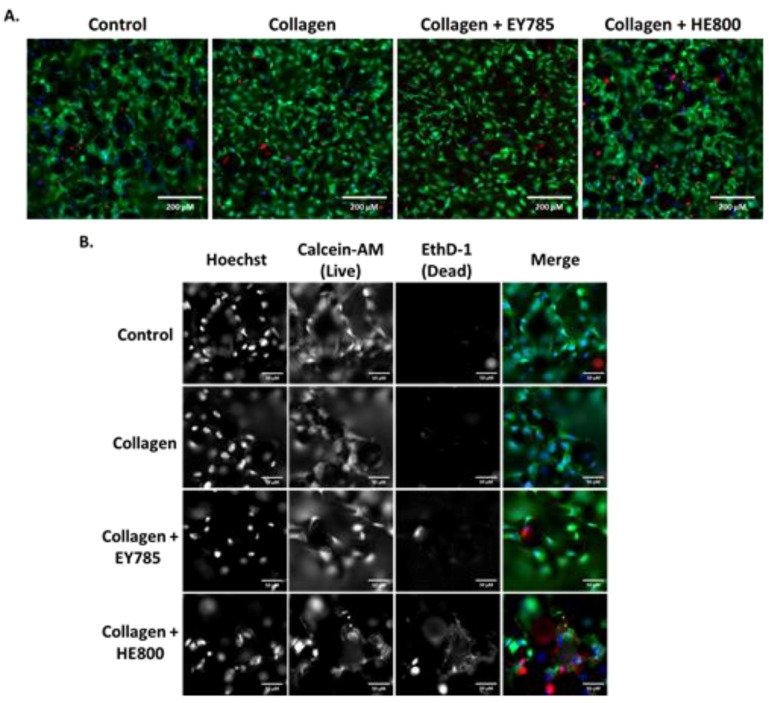
Fluorescence microscopy imaging of MG63 cells on Ti6Al4V samples after 7 days. Control: bare Ti6Al4V; blue: Hoechst staining; green: calcein-AM staining; red; Ethd-1 staining. (**A**) Images taken in widefield; (**B**) images taken at 5× magnification (with channels split). Scale bars: (**A**) 200 μm; (**B**) 50 μm.

The results of Live/Dead staining and fluorescence microscopy are shown in Figure 4. Cells on all sample groups exhibited a spread morphology, which is characteristic for good adhesion. Nearly all cells appeared viable. Only a few dead, red-stained cells were observed.

## 4. Discussion

In this study, SEM analysis (Figure 1) demonstrated the formation of collagen fibril coatings on Ti6Al4V surfaces. It appeared that the presence of anionic EPS derivatives increased the thickness of collagen fibrils, but this cannot be concluded conclusively from the SEM data. No definite influence of EPS derivatives on banding morphology was observed.

It was previously shown that the addition of the native HMW HE800 EPS during collagenous matrix remodeling increased the formation of *D*-periodic striated collagen fibrils [10]. In other previous studies, formation of fibrillar collagen in the presence of anionic polysaccharides, such as alginate or GAG (e.g., heparin and hyaluronic acid), has been reported [28,29,30]. The kinetics of collagen self-assembly and the fibril thickness were highly affected by the nature of the polysaccharide due to electrostatic interactions between negatively charged polysaccharides and positively charged regions on collagen. Different authors have reported that polysaccharides influence the thickness of collagen fibrils. For example, the presence of alginate led to an increase in collagen fibers [30]. However, other authors have reported that other polysaccharides, such as GAG, decrease fibril thickness. Addition of the GAG chrondroitin sulfate reduced the thickness of fibrils of collagen types I and II in previous work [6]. Sulfated hyaluronic acid led to a decrease in collagen fibril diameter [28]. 

AFM analysis of dried, highly-diluted EPS derivative solutions (Figure 2) revealed the formation of “threads” which are in a similar size range to the threads observed on SEM images (Figure 1). On both SEM and AFM images revealed that HE800 derivative formed longer threads than GY785 derivative. The threads were present at different points on the fibrils. In other words, threads did not appear to associate preferentially with any region of the fibril. It is not clear, from the results of this study, whether formation of the threads takes place prior to or after EPS derivatives bind to the surface of fibrils. The nature of the bonding between EPS derivatives and fibrils in this study is not clear. Non-covalent interactions, such as water-mediated hydrogen bonds, van der Waals forces, or electrostatic interactions between anionic polysaccharide chains and positive charges on the surface of the fibrils, can be expected to play a role.

In this study, no obvious advantage of the collagen fibril coating nor the presence of EPS derivatives on cell viability and morphology was demonstrated (Figure 3 and Figure 4). We decided to use the PrestoBlue cell viability reagent and Live/Dead staining, which have been used in hundreds of published studies to-date. The use of microscopy and Presto Blue has some limitations. We considered performing flow cytometry, but decided against it, because it would require cells to be removed from the EPS/collagen coated TiAl6V discs. Trypsinisation of cells where collagen is present could reduce the number of cells collected due to non-specific cleavage of proteins. In addition, increasing incubation times with trypsin may reduce cell viability.

It was previously shown that the addition of the native HMW HE800 EPS promoted human dermal fibroblast migration and proliferation [10]. Native HE800 and GY785 both promoted attachment of osteoblast and chondrocyte cell lines in previous work [31]. The derivatives used in this study are of smaller molecular weight. It has been reported that biological activity of polysaccharides, such as their effect on cell proliferation, can be affected by molecular weight [32].

Further work should focus on the use of primary cells, which may be more sensitive to differences in coating structure than cell lines. Osteogenic differentiation should also be investigated. From the point of view of physicochemical characterization of the coatings, it would be desirable to develop a technique to detect and quantify EPS chemically. There may be differences in coating thicknesses that might influence the results. Hence, it would be desirable to develop a technique to determine coating thickness.

## 5. Conclusions

Collagen fibril layers were formed successfully on Ti6Al4V discs produced by rapid prototyping. EPS derivatives were found associated with the fibrils on the coatings. Coatings did not markedly influence the attachment, morphology, and vitality of MG63 osteoblast-like cells cultured on the Ti6Al4V discs.

## Figures and Tables

**Table 1:** Osidic composition (wt%), sulfur content, S (wt%), and weight-average molecular weight, Mw (g/moL), of HE800 and GY785 derivatives.

Exopolysaccharides (EPS) Derivative	Osidic Composition (wt%)						S (wt%)	Mw (g/moL)
	Gal	Glc	GalA	GlcA	GalNAc	GlcNAc		
HE800 derivative	0	0	0	19.8	10.6	10.8	0	280 000
GY785 derivative	19.2	16.8	6.9	9.3	0	0	3	240 000

**Table 2:** The composition of the hydrogels produced. MEM–Minimum Essential Medium, ddH_2_O–double-deionized water.

Components	Volume, μL
Collagen Type I (4 mg/mL)	280
10× MEM	40
HE800/GY785 derivative (5 mg/mL ddH_2_O) or ddH_2_O	80
1 M NaOH solution	~30

## Supplementary Materials

The following are available online at http://www.mdpi.com/2072-666X/10/1/68/s1, Figure S1: Hydrogels formed by neutralization of acidic collagen solution containing exopolysaccharides (EPS). Left: hydrogel containing GY785; middle: hydrogel containing HE800; right: hydrogel without EPS.
