# Supplementary material for: Marine Polysaccharide-Collagen Coatings on Ti6Al4V Alloy Formed by Self-Assembly
Source: Micromachines (Basel). 2019 Jan 19;10(1):68. doi: 10.3390/mi10010068 (PMC6356479; doi:10.3390/mi10010068)
Supplement: Supplementary file 1 [file micromachines-10-00068-s001.pdf]

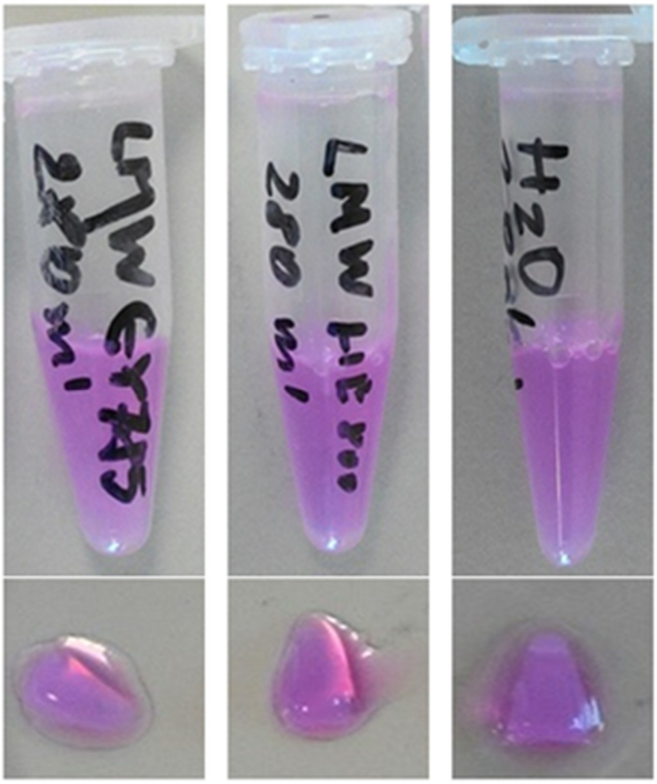

Figure S1. Hydrogels formed by neutralization of acidic collagen solution containing exopolysaccharides (EPS). **Left**: hydrogel containing GY785; **middle**: hydrogel containing HE800; **right**: hydrogel without EPS.
